# Association of hemispheric retinal oxygen extraction and macular plexus-specific capillary density in moderate to advanced glaucomatous hemifield defect

**DOI:** 10.1038/s41598-025-16127-w

**Published:** 2025-10-01

**Authors:** Martin Kallab, Nikolaus Hommer, Andreas Schlatter, Clemens Vass, René M. Werkmeister, Doreen Schmidl, Leopold Schmetterer, Gerhard Garhöfer

**Affiliations:** 1 Department of Clinical Pharmacology, Medical University of Vienna, Währinger Gürtel 18-20, Vienna, 1090 Austria; 2 Vienna Institute for Research in Ocular Surgery (VIROS), Hanusch Hospital, Vienna, Austria; 3 Department of Ophthalmology and Optometry, Medical University of Vienna, Vienna, Austria; 4 Center for Medical Physics and Biomedical Engineering, Medical University of Vienna, Vienna, Austria; 5 Singapore Eye Research Institute, Singapore National Eye Centre, Singapore, Singapore; 6 Academic Clinical Program, Duke-NUS Medical School, Singapore, Singapore; 7 SERI-NTU Advanced Ocular Engineering (STANCE), Singapore, Singapore; 8 School of Chemical and Biomedical Engineering, Nanyang Technological University, Singapore, Singapore; 9 Fondation Ophtalmologique Adolphe De Rothschild, Paris, France; 10 Aier Hospital Group, Changsha, China

**Keywords:** Retinal oxygen metabolism, POAG, Retinal oxygen extraction, Optical coherence tomography angiography, Retinal microcirculation, Optic nerve diseases, Medical research

## Abstract

The aim was to characterize associations of the hemispheric retinal oxygen metabolism with glaucomatous damage and with retinal plexus-specific microvascular information in 35 hemispheres of 25 primary open-angle glaucoma patients with moderate to advanced glaucomatous hemifield defect. Mean deviations (MDs) were calculated using retinal ganglion cell (RGC) density-weighted, perimetric total deviations. RGC counts were modelled from retinal nerve fiber layer thickness. Using single-vessel blood flow (Doppler-OCT) and spectroscopic oxygen saturation, retinal blood flow (RBF) and oxygen extraction (extO_2_) were calculated. From macular OCT-angiography superficial, intermediate and deep capillary densities (SCD, ICD and DCD) were extracted. In POAG, hemispheric positive associations of RBF and extO_2_ with structural (RGC) and functional (MD) glaucomatous damage were modest (r^2 ^ranging from 0.086 to 0.136, p-values between 0.076 and 0.029). Further analysis revealed that extO_2_ was more strongly associated with ICD and DCD (ICD: r^2^ = 0.274, *p* = 0.002, DCD: r^2^ = 0.190, *p* = 0.009) than with SCD (r^2^ = 0.141, *p* = 0.037). In contrast, no significant associations were observed between RBF and any macular capillary density across all layers (*p* > 0.05). ExtO_2_ variations appeared to reflect changes in deeper rather than superficial plexus capillary density, a relationship not observed for RBF. This suggests that RGC degeneration may lead to reduced oxygen consumption in the superficial retina accompanied by a relative increase in oxygen demand from the deeper retinal microvasculature.

## Introduction

Impairment of retinal oxygen metabolism has long been implicated in glaucoma pathogenesis and disease progression^1,2^. While it is still subject to scientific debate whether its alterations are causes or consequences of glaucomatous optic nerve head damage, non-invasive retinal oximetry and – in combination with retinal blood flow (RBF) measurement – oxygen extraction (extO_2_) measurements have been successfully deployed to study the retinal oxygen metabolism in glaucoma^3^. In this context, it was repeatedly shown that retinal venous saturation (higher) and/or arteriovenous saturation difference^4–11^ (lower), RBF^12–14^ (lower) and oxygen extraction^13,14^ (lower) are altered in glaucoma patients. There are, however, ongoing discussions whether alterations of the retinal oxygen metabolism in glaucoma correlate with structural and functional parameters of disease progression (i.e. retinal nerve fiber layer thickness and visual field defects)^4–6,15,16^ or not^17–19^. Moreover, all available non-invasive measurement techniques for retinal oxygen metabolism are restricted to the retinal surface while it has been shown that the plexuses of the retinal microcirculation react differentially to experimental variations in oxygen tension^20–24^. Plexus-specific information on correlations of the retinal oxygen metabolism with the retinal microcirculation is, however, currently lacking for glaucoma.

Using an approach to calculate retinal oxygen extraction in humans from Doppler optical coherence tomography (DOCT) derived RBF data and non-invasive retinal oximetry previously described by Werkmeister et al.^25^we could recently show that total retinal extO_2_ of the whole retina is significantly reduced in early glaucoma and correlates to structural and functional disease parameters^14^. This project focused on the later stages of POAG to determine whether retinal oxygen metabolism continues to correlate with functional and structural damage over the entire course of the disease. If these associations diminish, potential reasons for the shift will be explored.

We, therefore, refined our experimental approach to increase the granularity of our investigations to retinal hemispheres and made use of OCT angiography (OCTA)-derived depth-resolved retinal microvascular information.

Thus, the purpose of this study was to characterize the associations of the hemispheric retinal oxygen metabolism with structural as well as functional glaucomatous damage and with retinal plexus-specific microvascular information in POAG with moderate to advanced hemifield defect.

## Results

### Baseline characteristic

A total of 50 hemispheres of 25 POAG patients and 30 hemispheres of 15 healthy subjects were included. POAG patients and healthy subjects exhibited no significant differences in age and sex distributions (Table 1). Of the 25 POAG patients, 23 were receiving IOP-lowering medication with a median of two different agents per patient. These included 22 prostaglandin analogues, 15 beta-receptor-blockers, 11 carbonic anhydrase inhibitors and 6 alpha-2-agonists. Additionally, 6 patients had undergone filtration surgery at least six months prior to study inclusion. Further relevant baseline characteristics are summarized in Table 1.

**Table 1:** Baseline Characteristics.

	HS	POAG	*p* group difference
N	15	25	
Age	61.40 ± 7.21	65.12 ± 7.57	0.1341
Sex (male/female)	4/9	11/15	0.7397
SBP	130.3 ± 13.74	135.3 ± 13.35	0.2588
DBP	79.47 ± 9.91	77.08 ± 8.44	0.4223
MAP	100.8 ± 12.51	103.8 ± 11.89	0.4533
IOP	14.60 ± 1.99	13.92 ± 2.81	0.4178
RGC_WR_	1 076 084 ± 92 564	408 033 ± 143 636	**< 0.0001**
RGC_sup_ RGC_inf_	530 642 ± 54 463 545 442 ± 43 948	223 444 ± 111 297 184 589 ± 87 870	**< 0.0001** **< 0.0001**
MD_HFA_		-12.17 ± 5.77	
MD_sup_ MD_inf_		-14.02 ± 7.32 -11.67 ± 8.74	
MEDs (Median, min-max)		2, 0–4	
SURG (N of > = 1 SURG)		6	

HS: healthy subjects. POAG: primary open-angle glaucoma. RGC_WR_, RGC_sup_ and RGC_inf_: retinal ganglion cell count of the whole retina, superior and inferior hemisphere. MD_HFA_: MD as calculated by Humphrey Field Analyzer for 30 − 2 VF; MEDs: number of IOP lower medication; SURG: filtration surgery.

### Comparison of whole retinal and hemispheric RBF and extO_2_ between healthy subjects and POAG patients

Whole retinal RBF and extO_2_ in healthy subjects vs. POAG patients were 36.21 ± 5.72 vs. 26.79 ± 6.90 µl/min and 2.25 ± 0.60 vs. 1.89 ± 0.50 µl O2/min respectively and were found to be significantly different between healthy subjects and POAG patients. (*p* < 0.05 for all comparisons, Table 2).

**Table 2:** Whole retinal (WR) and hemispheric (SUP/INF) RBF and extO_2_ in healthy subjects (HS) and POAG patients.

	HS			POAG			*p* HS vs. POAG			*p* SUP vs. INF	
	WR	SUP	INF	WR	SUP	INF	WR	SUP	INF	HS	POAG
RBF (µl/min) Mean ±SD	36.21 ± 5.72	18.70 ± 4.87	17.51 ± 2.77	26.79 ± 6.90	14.84 ± 4.41	11.95 ± 3.25	**<** **0.0001**	0.0404*	**<** **0.0001**	0.7197*	**0.0004**
extO_**2**_ (µl O_2_/ min) Mean ± SD	2.25 ± 0.60	1.13 ± 0.38	1.12 ± 0.35	1.89 ± 0.50	1.03 ± 0.33	0.86 ± 0.25	**0.0484**	0.3729	**0.0099**	0.8756	**0.0126**

*Non-parametric test.

In healthy subjects superior / inferior RBF_hemi_ and extO_2,hemi_ were 18.70 ± 4.87 / 17.51 ± 2.77 µl/min and 1.13 ± 0.38 / 1.12 ± 0.35 µl O_2_/min. In POAG patients superior / inferior RBF_hemi_ and extO_2,hemi_ were 14.84 ± 4.41 / 11.95 ± 3.25 µl/min and 1.03 ± 0.33 / 0.86 ± 0.25 µl O_2_/min. Similarly to whole retinal values superior and inferior RBF_hemi_ as well as inferior extO_2,hemi_ were significantly different between healthy subjects and POAG patients (*p* < 0.05 for mentioned comparisons, Table 2) while differences in superior extO_2,hemi_ did not reach significance. Upon comparison of RBF_hemi_ and extO_2,hemi_ in healthy subjects, no significant differences between the superior and inferior hemisphere were found (*p* > 0.05 for both comparisons, Table 2), while hemispheric differences in POAG patients were statistically significant with higher values in the superior hemisphere (*p* < 0.05 for both comparisons, Table 2).

### Evaluation of associations

To analyze associations in moderate to advanced visual field defect only, hemifields >-6dB were omitted before further analysis. Therefore, a total of 35 hemispheres of 25 POAG patients (mean + SD MD: -16.45 ± 6.33) were included.

#### Associations of the retinal oxygen metabolism with hemispheric structure (RGC) and function (MD) in POAG patients

While positive associations of RBF_hemi_ and extO_2,hemi_ with MD_hemi_ were low but significant, their associations with RGC_hemi_ did not reach significance. (r^2^ between 0.182 and 0.033, *p* = 0.029–0.076). Details of these associations are summarized in Table 3.

**Table 3:** Associations with hemispheric structure (RGC) and function (MD) in POAG patients.

	b (95% CI)	*r* ^2^	*p*
RGC_hemi_			
RBF_hemi_	5723 (-59–11506)	0.105	0.052
extO_2,hemi_	64759 (-7237–136755)	0.086	0.076
MD_hemi_			
RBF_hemi_	0.56 (0.14–1.10)	**0.116**	**0.045** ^**1**^
extO_2,hemi_	7.55 (0.82–14.28)	**0.136**	**0.029** ^**2**^

b: unstandardized regression coefficient.

^1^Result from linear regression, LMM result after last iteration without convergence: b (95% CI): 0.56 (0.14–1.10), r^2^: 0.116, p:0.045.

^2^Result from linear regression, LMM result after last iteration without convergence: b (95% CI): 7.55 (0.82–14.28) r^2^: 0.133, p:0.029.

#### Associations of plexus specific capillary densities with hemispheric extO_2_ and RBF in POAG patients

**Fig. 1:**
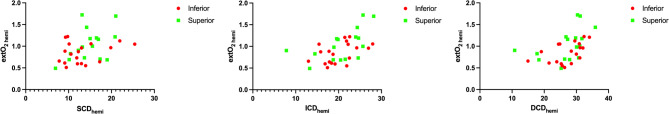
Scatterplots of associations between the retinal oxygen metabolism and retinal plexus-specific capillary densities. left: association of SCD_hemi_ and extO_2,hemi_. middle: association of ICD_hemi_ and extO_2,hemi_. right: association of DCD_hemi_ and extO_2,hemi_.

ExtO_2,hemi_ showed stronger positive associations with ICD_hemi_ and DCD_hemi_ (r^2^ = 0.274 and 0.190) than with SCD_hemi_ (r^2^ = 0.141) (Fig. 1). In contrast, no significant associations were observed between RBF_hemi_ and any hemimacular capillary plexus (r^2^ ranging between 0.035 and 0.085). Table 4 provides detailed information on these associations.

**Table 4:** Associations with hemispheric extO_2_ and RBF.

extO_2,hemi_	b (95% CI)	*r* ^2^	*p*	RBF_hemi_	b (95% CI)	*r* ^2^	*p*
SCD_hemi_	0.027 (0.002–0.052)	**0.141**	**0.037**	SCD_hemi_	0.240 (-0.62-0.541)	0.073	0.115
ICD_hemi_	0.036 (0.015–0.057)	**0.274**	**0.002**	ICD_hemi_	0.248 (-0.570-0.552)	0.085	0.107
DCD_hemi_	0.025 (0.007–0.042)	**0.190**	**0.009**	DCD_hemi_	0.130 (-0.110-0.370)	0.035	0.278

b: unstandardized regression coefficient.

## Discussion

In this study, we analyzed the hemispheric retinal oxygen metabolism and its depth-resolved association with the corresponding hemimacular microvascular bed in moderate to advanced glaucomatous hemifield defect. RBF and extO_2_ were significantly reduced in glaucoma patients as compared to healthy subjects. In patients with POAG associations of extO_2_ and RBF with structural (RGC count) and functional (MD) measures of glaucomatous damage were weak. Hemimacular capillary associations with extO_2_ were stronger in the deeper than in the superficial plexuses while no significant associations were observed for RBF. These findings suggest that, unlike RBF, extO_2_, is more strongly influenced by pathophysiological changes in the deeper retinal microvasculature in advanced stages of POAG.

Our finding of reduced RBF in POAG, which correlates with disease progression aligns with recent studies using DOCT to assess RBF in glaucoma^12–14^. To date, two studies have evaluated retinal oxygen extraction in POAG patients: Aref et al. reported significant associations of MD with RBF and oxygen extraction fraction but not with extO_2_^13^. In contrast, our previous work demonstrated strong and highly significant correlations of structural and functional glaucomatous damage with both RBF and extO_2_^14^. In the present study, however, we observed only weak associations for both RBF and extO_2_ with borderline statistical significance for functional loss and near-significant trends for structural damage. These discrepancies across studies may reflect differences in glaucoma severity among cohorts, as both Aref et al.’s (mean MD -13.76 dB) and our current cohort (mean MD_hemi_: -16.45 dB) represent more advanced disease stages. Although measurement floors are well-recognized for structural OCT parameters in advanced glaucoma^26 ^their potential influence on RBF and extO_2_ assessments remains underexplored and may contribute to the attenuated associations observed in more progressed disease. Similarly, differences in disease stages might also explain varying results in multiple previous studies on a potential dependency of retinal oximetric data (venous oxygen saturation, arteriovenous oxygen difference) on VF MD and or RNFL-T^4–6,15–19^. Finally, methodological differences regarding DOCT setups and oximetry approaches might be responsible for above-mentioned differences in study results.

To date, no studies on retinal oxygen extraction in glaucoma have incorporated OCTA-derived plexus specific macular microvascular data to generate hypotheses about potential pathophysiological mechanisms underlying the reduced associations of RBF and extO_2_ with disease progression in advanced stages. One OCTA study compared experimental hyperoxia-induced superficial peripapillary vessel density decreases between healthy subjects and POAG patients which were found to be diminished in POAG patients, however, no retinal oximetry was performed^27^. In another OCTA study on the peripapillary vessel density of the full thickness retinal vasculature at once, the OCTA parameter was found to be significantly correlated with retinal oximetric data (venous oxygen saturation, arteriovenous oxygen difference)^28^. The latter approach was suitable to confirm a basic relationship between oximetry and peripapillary OCTA-derived angiographic information, but did not provide depth-resolved plexus specific microvascular information. This, however, may be relevant in answering the question why retinal oxygen extraction (and retinal blood flow) in more progressed glaucoma stages appear to be poorly associated with structural and functional glaucomatous damage. Aref et al., in their above-mentioned study, speculated that upon impairment of choroidal blood flow^29,30^ compensatory oxygen supply by the retinal circulation to the outer retinal layers might occur. A loss of correlation between peripapillary retinal arterial and venous oxygen saturations and choroidal thickness in glaucoma patients as reported by Van Keer et al. might be indicative of disturbances of the shared retinal oxygen supply of the choroid and retinal vasculature in glaucoma^31^. We hypothesize that the stronger association between extO_2_ and ICD/DCD compared to SCD, alongside the lack of association between RBF and any hemimacular capillary density, provides initial clinical evidence supporting the theory of compensatory outer retinal oxygen supply by the retinal circulation in glaucoma. This suggests that the outer retinal layers experience a disproportionately increased oxygen demand that is not fulfilled by increased RBF but is instead met by an increase in DCD. Furthermore, the differential association patterns observed among the three macular capillary plexuses and extO_2_ imply a decreasing contribution of RGC oxygen demand to overall retinal oxygen consumption as RGCs progressively degenerate. While OCTA capillary density measurements do not allow for differentiation which vascular regulatory response takes place (diameter or velocity changes) in the above-mentioned scenario, hypoxia-induced autoregulatory vasodilation would, however, be pathophysiologically feasible. Using a similar OCTA based approach of depth-resolved analysis of the retinal microcirculation, we could recently show that DCP decreases upon experimental hypoxia challenge in healthy subjects^24^.

Our study has several strengths: Firstly, the novel anatomical matching approach of hemispheric oxygen metabolism parameters with macular plexus-specific microvascular information of the same hemisphere allows for the depth-resolved correlation of microvascular information and the oxygen metabolism in glaucoma. There are some previous studies investigating the hemispheric retinal oxygen metabolism in glaucoma^15,16,19^. Typically, these studies determined a better and worse hemisphere according to VF indices and compared oximetry values between them in every patient (paired analysis). We decided to take another approach because it is not dependent on large enough inter-hemispheric differences in glaucomatous damage and it makes analysis of associations between assessed variables possible. Regarding the latter, we could still include both hemispheres of patients into the same regression due to fitting of a linear mixed model, which accounted for non-independence of hemispheres within the same patient.

Secondly, the inclusion of glaucoma patients with hemifield MDs <= -6 dB and a mean MD of -16.45 dB made it possible to study the oxygen metabolism in advanced glaucoma stages. To ensure comparability of calculated MD_hemi_ with centrally-weighted whole visual field MD of the Humphrey Field Analyzer onboard analysis^32 ^TD values were locally weighted according to the physiological RGC density at the respective retinal location by using RGC density eccentricity curves from human post-mortem material published by Curcio et al.^33^.

Thirdly, with regard to DOCT, we relied on our previously developed dual-beam DOCT prototype, which was recently shown to have excellent repeatability and reproducibility^34^. Using this approach for absolute blood flow measurements in combination with oximetry we previously found retinal extO_2_ to be also reduced in type 1^35^ and type 2 diabetes^36^ as well as multiple sclerosis with history of optic neuritis^37^ and to be prone to a age-dependent decline^38^.

Finally, applying a model to calculate RGC counts from RNFL-T data made it possible to use a RNFL-T based structural glaucoma progression parameter correcting for age-related RGC density decline and changing proportion between axonal and non-axonal portion of the RNFL in the course of glaucoma progression; two factors that are usually left unaddressed when using RNFL-T as structural progression parameter. Our RGC count values in POAG patients are comparable with previous reports^39^.

Following limitations of our study ought to be mentioned: First of all and most importantly, due to its cross-sectional nature, our study was not designed to answer whether the encountered alterations in the oxygen metabolism are cause or consequence of glaucoma progression and all mentioned theories on explanations for the detected associations with the retinal microcirculation require confirmation in longitudinal clinical studies. Moreover, from a technical perspective, the OCTA signal is generated by decorrelation. Therefore, differences in vessel densities might be related to differences in blood velocities, diameter differences or degenerative capillary drop out, which should be kept in mind when interpreting OCTA data. Furthermore, the assessed parameters differ in spatial coverage and sensitivity to peripheral retinal changes. OCTA measurements were limited to a central 6° scan area, whereas both extO_2_ and RBF reflect global retinal values. Since RGC density is highest in the macula, retinal blood flow and oxygen extraction directed to the macula substantially exceed those in the peripheral retina. Finally, the sample size of our study was relatively small, partly due to the demanding nature of DOCT-based RBF measurements, which require high patient compliance. These examinations are particularly challenging for patients with pronounced VF defects who often struggle with fixation. To partially address this, we used a linear mixed model that allowed inclusion of both hemispheres of eyes separately in the regression analysis when applicable. However, due to the limited sample size, multivariate regression analysis was not feasible, preventing adjustment for potential confounders. We anticipate that future studies employing faster imaging algorithms in larger cohorts will overcome these limitations.

In conclusion, associations of retinal oxygen extraction and retinal blood flow with structural and functional glaucomatous damage are weak in moderate to advanced POAG. Notably, retinal oxygen extraction - but not retinal blood flow - variations appear to be driven by capillary density changes in the deeper rather than the superficial retinal plexuses. We hypothesize that reduced oxygen consumption in the superficial retinal layers due to RGC degeneration combined with alterations in choroidal oxygen supply may increase the relative oxygen demand of the outer retinal layers, which is then compensated by the deep retinal microcirculation. The results of this study are primarily of (patho)physiological and mechanistic interest. Nevertheless, retinal oxygen extraction may also serve as a potential biomarker for monitoring disease progression in late-stage glaucoma, although larger studies are needed to validate this hypothesis.

### Methods

#### Study design and subjects

This single-center cross-sectional study was performed at the Department of Clinical Pharmacology, Medical University of Vienna. Recruitment of glaucoma patients and healthy subjects took place between July 2018 and February 2021. Written informed consent was given by all subjects before inclusion and the protocol was approved by the Ethics Committee of Medical University of Vienna before initiation of the study. All study related procedures were performed in accordance with the Declaration of Helsinki and Good Clinical Practice guidelines of the International Council for Harmonisation.

### Inclusion and exclusion criteria

Inclusion criteria for glaucoma patients were: diagnosis of POAG, clinical glaucomatous optic neuropathy and hemispheric VF mean deviation (MD_hemi_, see below) of at least one hemisphere <= -6 dB. Exclusion criteria for glaucoma patients were: secondary glaucoma comprising pseudoexfoliation glaucoma and pigmentary glaucoma, angle closure glaucoma or history of acute angle closure, any other clinically significant ophthalmological disease, intraocular surgery < 6 months, untreated arterial hypertension (systolic blood pressure > 160mmHg and/or > 95mmHg), smoking, alcoholism and substance abuse, and ametropia > = 6 Dpt.

Inclusion criteria for healthy subjects were clinically insignificant medical history, clinically insignificant ophthalmological examination, and ametropia < 6 Dpt. Exclusion criteria were intraocular surgery < 6 months, untreated hypertension (systolic blood pressure > 160mmHg and/or > 95mmHg), smoking, alcoholism and substance abuse.

### Experimental paradigm

To evaluate eligibility a screening examination including documentation of medical history and concomitant medication, measurement of systemic haemodynamics, best-corrected visual acuity (BCVA), visual field (VF) testing using standard automated perimetry (SAP; Humphrey Field Analyzer HFA II 740-17324-4.2/4.2, 30 − 2 SITA-Standard, Carl Zeiss Meditec Inc., Dublin, Ireland) axial eye length measurement (IOL-Master, Carl Zeiss Meditec Inc.), ophthalmological examination including dilated funduscopy and applanation tonometry.

If eligible, the study eye was defined (if both eyes were eligible: the eye with more severely deviated VF) and study inclusion was confirmed. Before a 20-minute resting period for hemodynamic stabilization, tropicamide eye drops were administered to the study eye to ensure adequate pupil dilation.

First, a retinal vessel analyzer (RVA, Imedos Systems, Jena, Germany) was used to take a 50° optic nerve head-centered fundus image for O_2_ saturation measurements. Then, RBF in all major retinal vessels was measured using a prototype-stage dual-beam DOCT system, which is described in detail elsewhere^40,41^. Afterwards, a macular OCTA volume scan (20°x20°, 512 B-Scans, 5112 A-Scans/B-Scan) and a circumpapillary OCT ring scan (3.5 mm diameter) were acquired using a commercially available SD-OCT(A) device (Spectralis, Heidelberg Engineering, Heidelberg, Germany).

### Measurements

#### Systemic hemodynamics and intraocular pressure measurement

Systemic hemodynamics were assessed using an upper arm automated oscillometric instrument (Infinity Delta; Dräger, Vienna, Austria). Systolic blood pressure (SBP), diastolic blood pressure (DBP), mean arterial pressure were measured.

Intraocular pressure (IOP) was assessed using Goldmann applanation tonometry after administration of one drop of oxybuprocainhydrochloride + sodium fluorescein.

#### Retinal ganglion cell density corrected Hemifield mean deviations

**Fig. 2:**
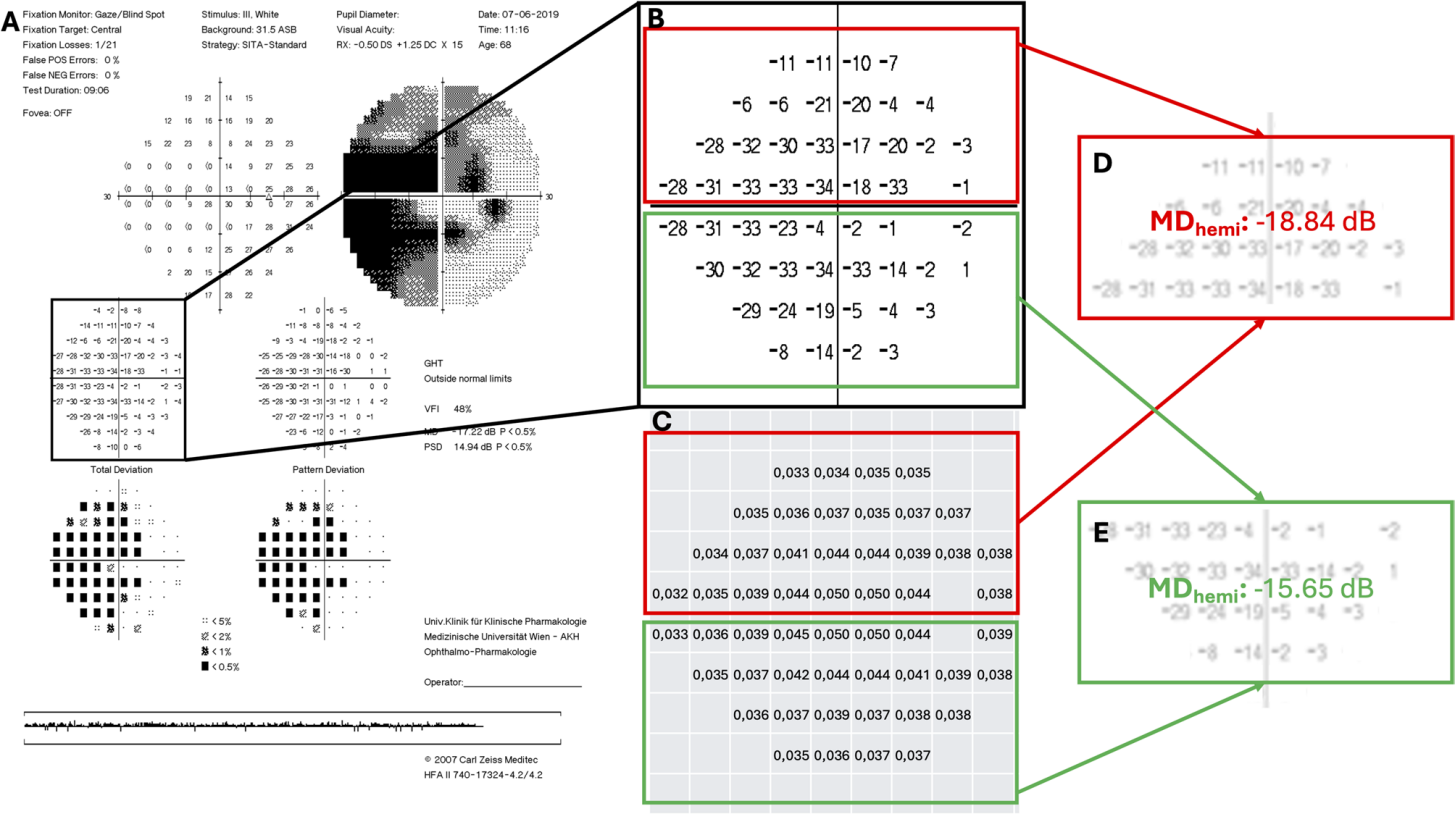
Retinal ganglion cell density corrected hemifield mean deviations. (**A**) Single Field Analysis (SFA) as exported using Humphrey on-board software. (**B**) Total Deviation Map from SFA. (**C**) weighting factors as calculated using local RGC densities. (**D**) superior MD_hemi_ as calculated by the weighted mean of all superior total deviation values. (**E**) inferior MD_hemi_ as calculated by the weighted mean of all superior total deviation values.

Raw total deviation values of all visual field test spots were extracted from “Single Field Analysis” printouts of SAP measurements for the inferior and superior hemifield separately (Fig. 2A) and outer superior, inferior and temporal test spots were omitted resulting in 24 − 2 total deviation maps of the superior and inferior hemisphere (Fig. 2B).

Then, raw total deviation values were weighted according to the physiological RGC density at the respective retinal location: Using RGC density eccentricity curves from human post-mortem material published by Curcio et al.^33^ every VF test spot was assigned to a weighting factor calculated from the logarithm of the weighted mean of the RGC density at the corresponding horizontal and vertical retinal position (Fig. 2C). Superior and inferior mean deviations (MD_sup_ and MD_inf_) were calculated as the weighted mean of all superior (Fig. 2D) and inferior (Fig. 2E) total deviation values, resembling the centrally-weighted Humphrey Field Analyzer on-board algorithm of MD calculation for the whole VF^32^.

#### Total and hemispheric retinal blood flow and oxygen extraction

Total RBF as well as hemispheric (i.e. superior and inferior hemiretinae separated by a papillo-macular line) RBF (RBF_hemi_) of the superior and inferior hemisphere were calculated based on measurements acquired with the above-mentioned DOCT-device. With the exception of separating peripapillary arteries and veins according to their hemispheric location for RBF_hemi_, RBF calculation was performed as described by Doblhoff-Dier et al.^41^ In short, various horizontal and vertical DOCT B-scans were acquired around the optic nerve head until major retinal vessels down to a diameter of 40 μm were included in the analysis.

An in-depth technical description of the dual-beam DOCT approach for velocity measurement in single retinal vessels used in our device is published elsewhere^40^. In short, the angle under which the two probe beams reach the retinal vessels, i.e. the Doppler angle, is unknown, however, the angle difference Δα between the two probe beams can be calculated taking the subject’s axial length and the beam separation at the pupil into account. Based on the OCT data of the separate channels, the difference (ΔΦ) of the phase shifts introduced in the two beams once reflected by erythrocytes can be calculated. Together with angle β between the plane spanned by the two beams and the vessel velocity vector as well as constants defined by the optical setup and the acquisition protocol, absolute velocity of blood (V_abs_) in a single retinal vessel can be calculated as described in Eq. (1).

1 $$\:{V}_{abs}={\Delta\:}{\Phi\:}\text{*}\frac{\lambda\:}{4\pi\:*n*\tau\:*{\Delta\:}\alpha\:*\text{cos}\beta\:}$$

Absolute blood flow (Q) can then be calculated using the vessel diameter D as estimated from the OCT phase tomogram and V_abs_ as described in Eq. (2).

2 $$\:Q=\frac{{D}^{2}}{4}*{\pi\:*V}_{abs}$$

For Doppler OCT (DOCT) measurements, participants stabilized their head using a chin and forehead rest and fixated on a movable target to aid alignment. The B-scan orientation could be rotated by 90°, allowing both horizontal and vertical scans. This flexibility enabled imaging of all major retinal vessels around the optic nerve head, facilitating accurate quantification of total retinal blood flow.

**Fig. 3:**
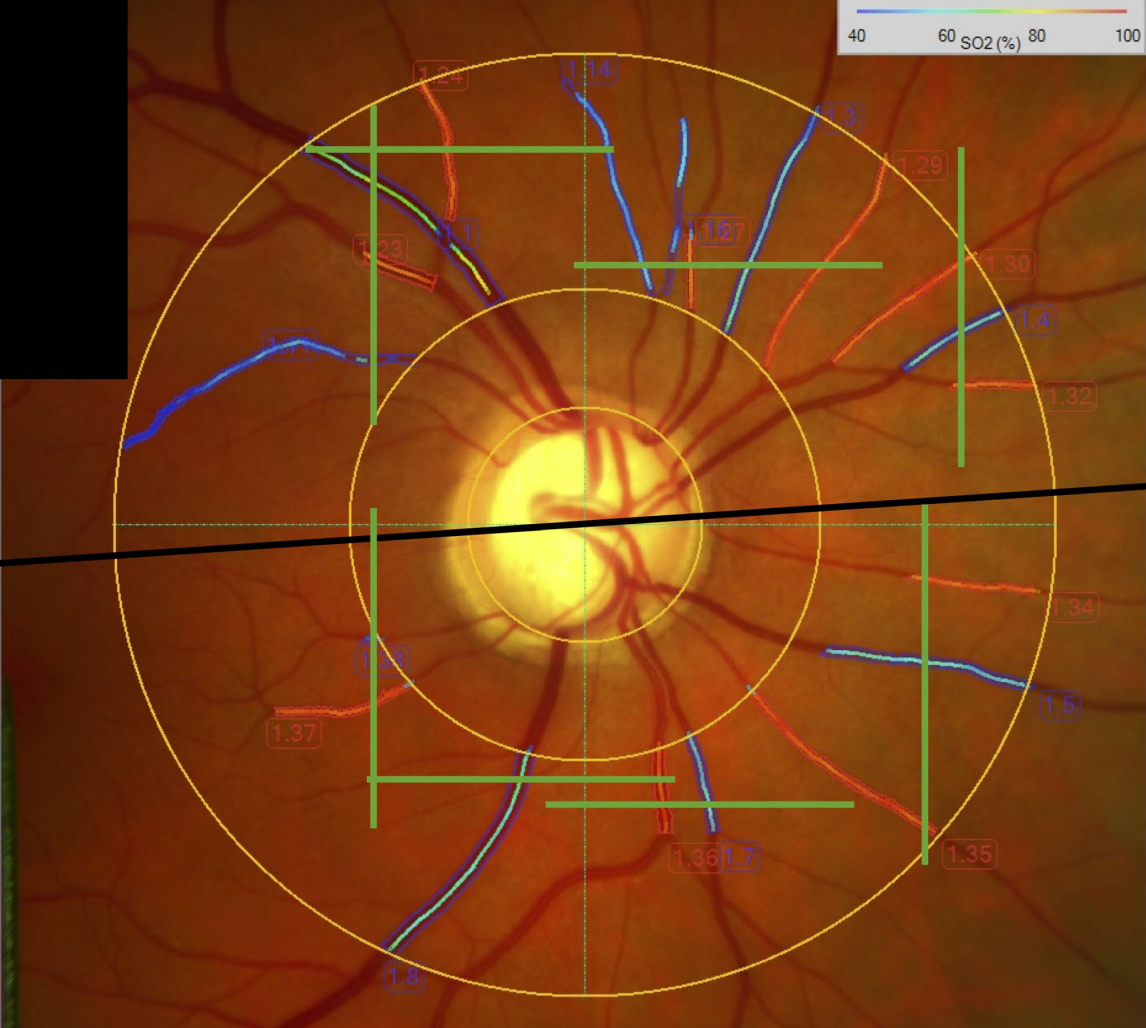
Oximetry and DOCT-based blood flow measurements for calculation of oxygen extraction. Fundus photograph with venous (blue) and arterial (red) overlay indicating the area of reflectometric oximetry. The green overlays indicate the areas of DOCT-based vessel-by-vessel blood flow measurement. The black line indicates the line of separation between superior and inferior hemispheres: Single-vessel oxygen saturations and blood flow values above this line were used to calculate extO_2,hemi_ of the superior hemisphere, Single-vessel oxygen saturations and blood flow values below this line were used to calculate extO_2,hemi_ of the inferior hemisphere.

Based on reflectometric estimation of oxygen saturation in retinal vessels from fundus images, which makes use of oxygenation-dependent light absorption profiles of the hemoglobin molecule^42,43^ and single vessel flow calculation (see Eq. 2) retinal oxygen extraction (extO_2_) can be calculated based on a model published by Werkmeister et al.^25^ In short, this model corrects for the distance of O_2_ saturation measurement to the central retinal vessel branching and accounts also for the plasma dissolved portion of blood oxygen. As a novelty and divergent to the published model, here we did not only calculate whole retinal extO_2_ but, similarly to calculation of RBF_hemi_ peripapillary arteries and veins were separated according to their hemispheric location (superior and inferior), resulting in extO_2,hemi_ values of the superior and inferior hemisphere (Fig. 3). All other model assumptions were identical.

#### Hemispheric macular plexus-specific capillary density

**Fig. 4:**
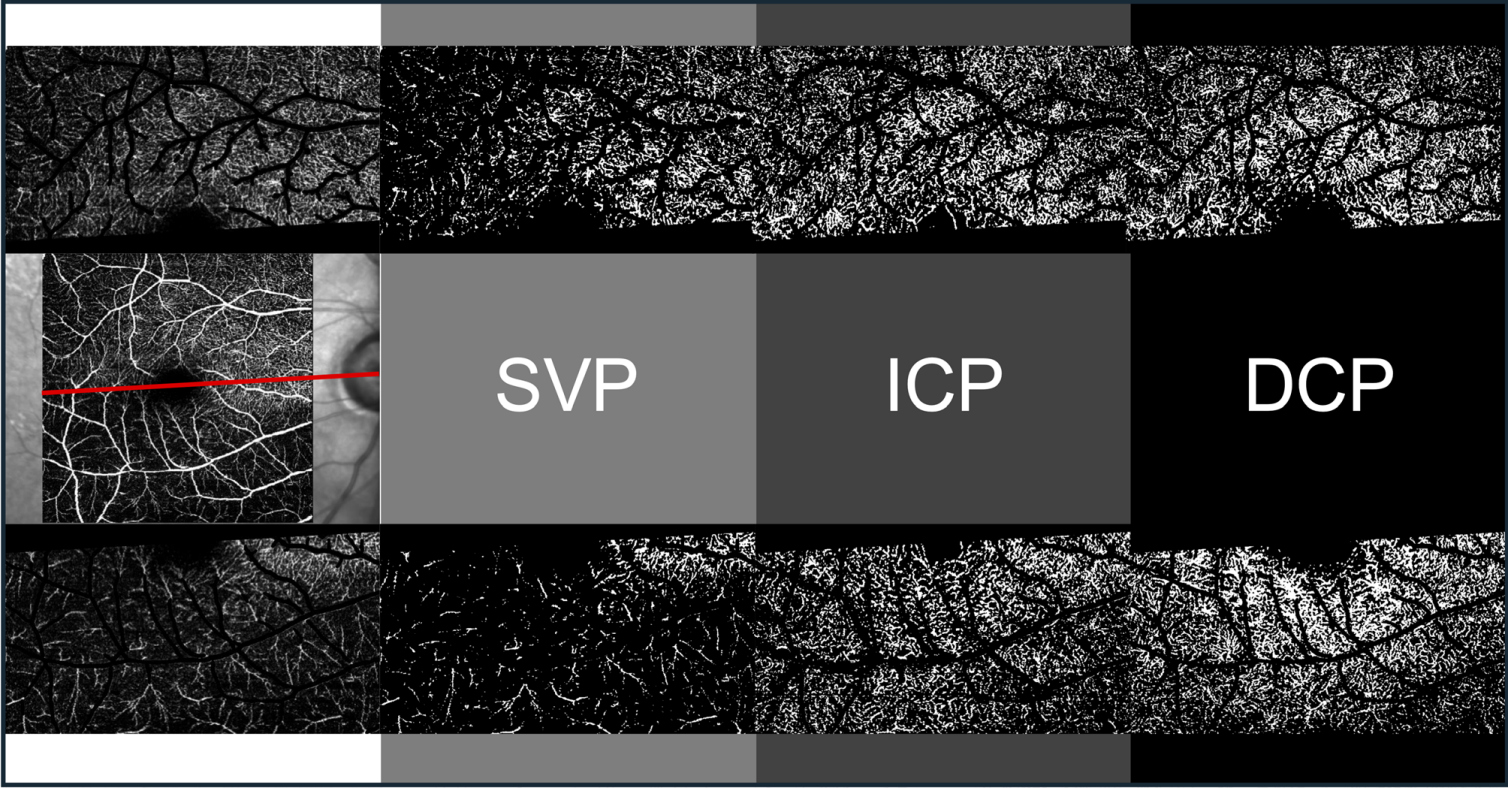
Representative OCTA slab processing workflow. white column: macular slabs were divided into an infero-macular und supero-macular portion and large vessels were removed using a Hessian-based large vessel removal filter. light grey column: binarized hemispheric superficial vascular plexus (SVP) slabs after large vessel removal. dark grey column: binarized hemispheric intermediate capillary plexus (ICP) slabs after projection artifact removal. black column: binarized hemispheric deep capillary plexus (DCP) slabs after projection artifact removal.

Macular OCTA slabs of the superficial vascular plexus (SVP), intermediate capillary plexus (ICP) and deep capillary plexus (DCP) were exported to Fiji ImageJ^44^ using standard slab and processing settings of the Spectralis OCTA module. Macular slabs were divided into an infero-macular und supero-macular portion by a line crossing the optic nerve head and the central avascular zone using a stitched overlay of the OCTA slab and a larger 30°x30° infrared image generated by the Spectralis device, in which the optic nerve head was visible. Afterwards large vessels in the SVP-slab and large vessel projection artifacts in the ICP and DCP were removed using a Hessian-based large vessel removal filter as previously published by our group^37^. Finally, images were binarized using the Otsu thresholding algorithm^45^ and hemispheric capillary densities of SVP (SCD_hemi_), ICP (ICD_hemi_) and DCP (DCD_hemi_) were calculated as the percentage of white pixels. Figure 4 summarizes relevant steps of OCTA image processing.

#### Hemispheric retinal ganglion cell count

Based on sectorial^46^ retinal nerve fiber layer (RNFL) thickness (RNFL-T) values extracted from standard 3.5 mm diameter circumpapillary OCT ring scans RGC count was estimated according to a model introduced by Harwerth et al.^39^ correcting for age-related RGC density (d) decline (Eq. 3) and changing proportion between axonal and non-axonal portion of the RNFL in the course of glaucoma progression (Eq. 5).

3 $$\:d=\left(0.007*age\left[years\right]\right)+1.4$$

Equation 4 shows the calculation of the raw sectoral RGC count (rsRGC) in one sector, where mt is the mean RNFL-T in µm, px is the length of the sector in pixels, 14.32 is the device OCT specific length per pixel in µm and d is the age dependent RGC density.

4 $$\:rsRGC=mt*px*14.32*d$$

To account for the above-mentioned changing proportion between axonal and non-axonal portion of the RNFL in the course of glaucoma progression, a correction factor c, which is dependent on the MD in the respective GH-sector was introduced (Eq. 5) and the logarithmic corrected sectorial RGC count (lsRGC) was calculated using rsRGC and c as shown in Eq. (6).

5 $$\:c=\left(-0.26*MD\right)+0.12$$

6 $$\:lsRGC=\left(\text{log}\left(rsRGC\right)*10\right)-c$$

After antilog and summation of all superior or inferior sectorial lsRGC counts hemispheric RGC count (RGC_hemi_) was calculated.

### Statistical analysis

Unless stated otherwise values are presented as means ± standard deviation (SD). Normality was assessed using the Kolmogorov-Smirnov test. For comparisons between healthy subjects and POAG patients unpaired t-tests or nonparametric equivalents were applied. Paired t-tests or nonparametric equivalents were used to evaluate hemispheric differences within subjects. Sex distribution was compared using Fisher’s exact test. To account for within-subject correlations when analyzing hemispheric associations, linear mixed models were employed with the subject treated as a random effect and the variable of interest as a fixed effect. Associations were reported using regression coefficients, 95% confidence intervals, p-values and marginal pseudo-R^2^ values. In cases of model non-convergence results from the final iteration of the mixed model and corresponding standard linear regression were reported. Statistical analyses involving linear mixed models were conducted using IBM SPSS Statistics (IBM Corp., Armonk, NY, USA). All other descriptive statistics, hypothesis testing and graphical outputs were generated using GraphPad Prism (GraphPad Software Inc. CA, USA). A p-value < 0.05 was considered statistically significant.

## Data Availability

The datasets generated during and/or analyzed during the current study are available from the corresponding author on reasonable request.
